# A program of SUPPORT-D^TM^: Feasibility and acceptability of an early palliative care intervention for those living with dementia and caregivers

**DOI:** 10.1017/S1478951525101429

**Published:** 2026-01-07

**Authors:** Diana Layne, Teresa Kelechi, Nicholas Milano, Mohan Madisetti, Kathleen Lindell

**Affiliations:** 1 College of Nursing, Medical University of South Carolina, Charleston, SC, USA; 2 College of Medicine, Medical University of South Carolina, Charleston, SC, USA

**Keywords:** Palliative care, Alzheimer’s disease, dementia, nurse-led intervention, caregiver

## Abstract

**Objectives:**

Persons living with dementia (PLWD) and their caregivers (CG) face a complex disease trajectory, which includes a multitude of challenges related to identifying credible health resources, access to services, and securing emotional support. Scalable, sustainable interventions that guide recently diagnosed PLWD and CG are desperately needed to minimize unnecessary burden and improve quality of life. This article describes the feasibility and acceptability of an early virtual palliative care intervention (SUPPORT-D^TM^) for use among PLWD with mild Cognitive Impairment or Alzheimer’s disease and their CG.

**Methods:**

Using a quasi-experimental design, this 6-week prospective feasibility study was conducted among 28 (PLWD/CG) dyads and 2 individual CG. Eligibility criteria for PLWD included those with mild cognitive impairment (FAST score ≥4). SUPPORT-D^TM^ comprises 4 main areas of guided support: 1) understanding the disease, 2) caring for myself, 3) caring for the caregiver, and 4) planning for the future. Outcome data were collected pre/post and during the intervention. Semi-structured interviews were conducted post intervention with 10 dyads. This study was approved by the Medical University of South Carolina IRB and data were collected from January 2023 to March 2024.

**Results:**

Seventy-six percent (23/30) of enrolled dyads successfully completed the study. PLWD and CG scores on validated measures of acceptability, appropriateness, and feasibility indicated SUPPORT-D^TM^ was acceptable, appropriate, and feasible. Post-intervention interview feedback further evidenced the acceptability, appropriateness, and feasibility of SUPPORT-D^TM^.

**Significance of results:**

Delivery of this virtual nurse-led early palliative care intervention (a Program of SUPPORT-D^TM^) was feasible for both PLWD and their CGs. A Program of SUPPORT-D^TM^ has potential as a feasible intervention to provide anticipatory guidance to community-dwelling PLWD and CG. Participants endorsed inclusion of additional content specific to physical activity, stress management, and social support as helpful refinements for future delivery.

## Background

Dementia caused by neurodegenerative diseases such as Alzheimer’s disease is an irreversible condition defined as an acquired loss of cognition in multiple cognitive domains sufficiently severe to affect social or occupational function (Arvanitakis et al. 2019). Alzheimer’s disease and related dementias (ADRD) typically progress from mild cognitive impairment early in the disease trajectory to late-stage dementia toward the end of the illness trajectory. Primary treatment goals for this chronic condition are to decrease suffering caused by cognitive and behavioral changes and delay inevitable progressive decline (Arvanitakis et al. 2019). This population experiences a higher burden of comorbid physical disease, polypharmacy (Clague et al. 2017) and, in advanced stages, challenges with independent mobility, increasing falls risk (Van Ooteghem et al. 2019). The variable and often prolonged illness trajectory coupled with progressive cognitive function decline makes symptom self-management complex often requiring assistance.

It is estimated that over 11 million Americans are providing unpaid care to persons living with dementia (PLWD) (Alzheimer’s Association 2024). Caregivers (CG) of PLWD report needing support to manage stress related to caregiving responsibilities, identifying respite care, building a network of support, and managing behavioral symptoms (Jennings et al. 2017). Many PLWD/CG dyads experience unmet needs which vary based on severity of cognitive impairment of the PLWD (Black et al. 2013; Jennings et al. 2017). PLWD and their CGs could benefit from interventions for improving early access to palliative care (Murphy et al. 2016). Palliative care is defined as specialized medical care for those living with serious illness such as dementia to improve quality of life for both the patient and family (Center to Advance Palliative Care 2024).

Despite a recent increase in hospice enrollments for PLWD (fewer than 1% in 1995 to 18% in 2017), challenges remain with palliative care, in particular, advance care planning (ACP) (Hashimie et al. 2020). Early ACP prior to the onset of severe cognitive impairment promotes goal concordant care and minimizes caregiver burden and stress during the end of life phase of the illness. Two identified barriers to early ACP initiation include a lack of understanding the disease trajectory and difficulty planning for an uncertain future (Tilburgs et al. 2018). Caregiver education regarding prognosis of dementia and ACP is limited (Stewart and Schultz 2018; Kim et al. 2021), which adds to the difficulties of early ACP in PLWD. The progression of the illness, which often renders PLWD unable to communicate their preferences, combined with an extended end-of-life phase, further inhibits the timely initiation of palliative care (Fox et al. 2017). As a result, palliative care interventions for PLWD and their CG are limited and are predominantly introduced during the later stages of ADRD (Butler et al. 2020).

To address the lack of palliative care interventions for recently diagnosed patients and CG, a nurse led early palliative care intervention entitled A Program of SUPPORT-D^TM^ (Symptom management, Understanding the disease, Putting safety first, Palliative care, Ongoing Conversations, Respite care and advanced Treatments) was adapted from A Program of SUPPORT^TM^ which is designed for those with pulmonary fibrosis and their CG (Lindell et al. 2021) for use among mildly cognitively impaired PLWD and their CG. The purpose of this study was to examine and report on the feasibility and acceptability of SUPPORT-D^TM^ for PLWD and CG.

## Methods

Following approval by the Medical University of South Carolina (MUSC) Institutional Review Board (PRO 00120938), a quasi-experimental pre/post design was used to assess feasibility and acceptability of SUPPORT-D^TM^ with PLWD and CG dyads. Participant dyads were recruited through various methods including clinic referrals from a local memory clinic and internal medicine clinic, local early memory loss programs, and advertisement on the Alzheimer’s Association and National Family Caregiver Alliance websites and social media. Participants were eligible if they were greater than 18 years old, able to read and speak English (intervention materials were in English), diagnosed with mild cognitive impairment, Alzheimer’s disease, or suspected Alzheimer’s disease with a Functional Assessment Score (FAST) (Sclan and Reisberg 1992) greater than or equal to 4 for PLWD. The CG were excluded if that had a diagnosis of cognitive impairment or were providing paid care to someone living with Mild Cognitive impairment, Alzheimer’s disease, or suspected Alzheimer’s disease who met eligibility requirements. CG were permitted to participate independently in the event the PLWD for whom they cared met inclusion criteria but declined to participate. Individuals unwilling to provide informed consent were excluded from study participation.

## Intervention

“A Program of SUPPORT-D^TM^” is a multicomponent, nurse led intervention booklet adapted from “A Program of SUPPORT^TM^” designed for those living with pulmonary fibrosis and their CG (Lindell et al. 2021). Specific details of the adaptation are described elsewhere (Layne et al. 2024). The intervention materials are copyright registered for intellectual property protection though the Medical University of South Carolina. Broadly, the content within SUPPORT-D^TM^ focuses on providing anticipatory guidance across 4 main topics: 1) understanding the disease, 2) caring for myself, 3) caring for the caregiver, and 4) planning for the future. Table 1 describes content for each component.

**Table 1.** SUPPORT-D component delivery and timing

Delivery timing	Intervention component	Rationale	SUPPORT-D component
Week 1–2	Part 1: Education regarding disease, illness trajectory	Patients/CGs are unaware of disease trajectory, prognosis	Understanding the disease
Week 1–2	Part 2: Self-management training for common challenges and strategies for safety	Progressive escalation of symptoms (e.g., anxiety, depression, poor sleep) and potential for unsafe situations as disease progresses (e.g., driving, financial safety)	Symptom Management, Putting Safety First, Ongoing Conversations, Alternative Treatments
Week 3	Nurse interventionist visit discussing parts 1 and 2		
Week 4–5	Part 3: Caring for CG	CG face tremendous burden and are often unprepared and neglect own needs due to demands of caregiving.	Putting Safety First, Ongoing Conversations, Respite Care
Week 4–5	Part 4: Planning for future including end-of life goal development	Lack of discussion among patient/CG prior to cognitive deterioration results in insufficient preparation for EOL decision making.	Palliative Care
Week 6	Nurse interventionist visit discussing parts 3 and 4		

## Study format and schedule

Once participant dyads were recruited, screened for eligibility, and informed consent completed, an orientation meeting with the study coordinator occurred to ensure dyads could access and operate MS Teams in preparation for 2 virtual meetings with the nurse interventionist. Consented dyads were provided a copy of the SUPPORT-D booklet along with access to a digital version which could be shared with other family members/friends. Dyads were instructed to review the first 2 parts of the SUPPORT-D booklet during the first 2 weeks and the last 2 parts during weeks 4 and 5. Dyads met with a nurse interventionist to discuss booklet contents based on individualized needs during weeks 3 and 6. Table 2 provides a description of intervention components including delivery timing.

**Table 2.** Demographic information by role

	Care recipient (*n* = 27)	Caregiver (*n* = 29)
Average age (SD)	75.1 (8.3)	69.5 (10.3)
Average household size (SD)	2 (0.7)	2 (0.6)
	**Frequency (%)**	**Frequency (%)**
**Gender**		
Male	19 (70%)	6 (21%)
Female	8 (30 %)	23 (79%)
Prefer not to say	0 (0 %)	0 (0%)
**Marital status**		
Never married	0 (0%)	1 (3%)
Married	24 (89%)	27 (93%)
Widowed	1 (4%)	0 (0%)
Separated	0 (0%)	0 (0%)
Divorced	2 (7%)	1 (3%)
**Race**		
American Indian or Alaskan Native	0 (0%)	0 (0%)
Asian	0 (0%)	0 (0%)
Native Hawaiian or Pacific Islander	0 (0%)	0 (0%)
Black or African American	1 (4%)	3 (10%)
White	26 (96%)	25 (86%)
Other	0 (0%)	1 (4%)
**Ethnicity**		
Hispanic or Latino	2 (7%)	2 (7%)
Not Hispanic or Latino	25 (93%)	27 (93%)
**Education**		
Some high school	0 (0%)	1 (3%)
High school graduate	5 (18.5%)	1 (3%)
Some college	5 (18.5%)	5 (17%)
College graduate	7 (26%)	10 (34%)
Post graduate degree and/or higher	10 (37%)	12 (41%)
**Household income**		-
$15,000–49,999	5 (20%)	
$50,000–99,999	4 (17%)	
$100,000–$150,000	6 (25%)	
$150,000 or greater	6 (25%)	
Unknown	3 (13%)	
**Relationship to care recipient**	-	
Spouse or partner		23 (79%)
Child		4(14%)
Other		2 (7%)
**Employment**	-	
Unemployed		1 (3%)
Retired		18 (62%)
Part-time		3 (10%)
Full-time		6 (21%)
No response		1 (3%)
**Served as caregiver previously**	-	
Yes		14 (48%)
No		15 (52%)

## Data collection

Demographic data and baseline outcome measures including disease specific knowledge, stress, symptom burden, disease related quality of life for the PLWD, caregiver self-efficacy for the CG, home safety. Additionally, technology literacy for PLWD was assessed at week 6. Data were stored within REDCap (Harris et al. 2009). Weekly surveys were also completed to report time spent reviewing intervention materials, difficulty accessing the electronic version of intervention materials or challenges with technology that occurred during the prior week. Outcome measures were collected post intervention along with acceptability, appropriateness, and feasibility measures. Finally, 10 dyads expressed willingness to participate in a post intervention interview to elicit data regarding intervention feasibility, acceptability and suggested modifications to intervention content or delivery methods.

## Caregiver instruments

### Caregiving burden

The brief Zarit Burden Interview (ZBI-12) was administered for CG to complete preintervention and post intervention. The ZBI-12 is a 12-item scale assessing role strain and personal strain. Responses are scored based on frequency of experienced feelings of burden from 0 (never to) to 4 (nearly always). A total score is calculated by summing all items with higher scores indicating greater burden (Bédard et al. 2001). The ZBI-12 demonstrates adequate internal consistency (Cronbach’s alpha = 0.88) (Bédard et al. 2001).

## Self-efficacy

The Self-Efficacy for Caregiving 8-Item scale (CSES-8) was used to measure caregiver self-efficacy. This scale includes 8-items to evaluate confidence in completing caregiving tasks regularly at the present time. Responses range from 0 (not at all confident) to 10 (totally confident), a mean total score is calculated across all items with higher scores indicating greater self-efficacy. The CSES-8 demonstrated acceptable internal consistency (Cronbach’s alpha = 0.88–0.89) and satisfactory construct validity (Kahle-Wrobleski et al. 2016; Ritter et al. 2022). Prior work indicates that self-efficacy change of 0.5 standard deviation unit is clinically meaningful (i.e., a Cohen’s d effect size of 0.5) (Ritter et al. 2022).

## Safety

The Safety Assessment Scale for People Living with Dementia Living at Home (SAS) was administered for CG to complete based on the home environment of the PLWD. The SAS is a 32-item scale assessing accident risk across 9 domains including caregiver presence, smoking, fire and burns, nutrition, food poisoning and toxic substances, medication and health problems, wandering and changing to adapting temperature, injuries, and driving. Responses are scored based on severity of potential risk, with “yes” or “no” responses receiving a score of 1 or 0, respectively, and responses with 3 or 4 choices per question scored from 1 to 4 based on degree of risk (higher scores indicate elevated accident risk). A total score is calculated by summing all items. Acceptable criterion and construct validity were demonstrated with satisfactory test-retest reliability (ICC = .9) and inter-rater reliability (ICC = .88) (de Courval et al. 2006).

## PLWD instruments

### Digital health literacy

The Digital Health Literacy instrument is a 21-item scale with responses on a 4-point Likert scale ranging from “very easy” (1) to “very difficult” (4). All items are reverse-coded prior to summing items for a total score. Subscale scores for operational skills, navigation skills, information searching, evaluating reliability, determining relevance, adding content, and protecting privacy can also be calculated by averaging scores across applicable items (van der Vaart and Drossaert 2017). This instrument has acceptable internal consistency (Cronbach’s alpha = 0.87) for overall digital health literacy and subscale internal consistency (Cronbach’s alpha = 0.70–0.89).

## Instruments for both CGs and PLWD

### Knowledge

The Alzheimer’s Disease Related Knowledge Scale (ADKS) was administered at baseline and post intervention. This 30-item dichotomous True/False survey was designed for use with students, healthcare professionals, and the public (ADKS | Clinical Geropsychology Laboratory | Washington University in St. Louis n.d.). Completion time is approximately 5–10 min and topics assessed include risk factors, assessment and diagnosis, symptoms, course, life impact, caregiving, treatment, and management. Furthermore, the ADKS has demonstrated content, predictive, concurrent, and convergent validity with satisfactory reliability (Cronbach’s alpha = 0.80). A total score is calculated using a summed total of correct items; higher scores indicate greater disease specific knowledge (Carpenter et al. 2009).

## Perceived stress

The Perceived Stress Scale (PSS) was administered at baseline and post intervention. This 10-item survey with Likert responses from “Never” (0) to “Very Often” (4) examines participants’ feelings and thoughts over the prior month. Completion time is approximately 5–10 min, and a summed score is used across all items, with scores ranging from 0 to 40, higher scores indicate greater perceived stress. Evidence suggests the PSS is a valid and reliable instrument (Cronbach’s alpha = 0.84–0.86) for measuring general stress (Cohen et al. 1983, c). Estimates of minimal clinically important difference were previously identified as 11 points (Eskildsen et al. 2015).

## Symptom burden

Symptom burden was measured utilizing Patient-Reported Outcomes Measurement Information System 29-Item Profile Measure (PROMIS-29 V2.0). These 29 items measure 8 domains including physical function, anxiety, depression, fatigue, sleep disturbance, ability to participate in social roles and activities, pain interference, and pain intensity. Responses range from “not at all” to “very much” and are scored from 1 to 5, respectively. Additional scoring information is available at http://www.healthmeasures.net/. Reliability for these measures ranges from 0.77 to 0.96. Prior work supports a change of 0.5 standard deviation unit change in PROMIS scale score T-scores as minimal clinically important difference (Hays et al. 2018; Khutok et al. 2021).

## Disease related quality of life for both CG and PLWD

The Quality of Life in Alzheimer’s disease (QOL-AD) scale participant version was used for self-assessment (PLWD) and proxy assessment (CG). Both versions of the QOL-AD include 13 items rating various aspects of quality of life. Scores are summed across all 13 items and range from a total score of 13–52 with higher scores indicating better quality of life. Cronbach’s alpha ranges from 0.74 for patients and 0.86 for proxies with satisfactory construct validity (Torisson et al. 2016). Prior research suggests a 3 point change in scores as a clinically important difference.

## Acceptability, feasibility, and appropriateness

The Acceptability of Intervention Measure (AIM), Intervention Appropriateness Measure (IAM), and Feasibility of Intervention Measure (FIM) were used to measure acceptability, feasibility, and appropriateness of the SUPPORT-D intervention. The AIM, IAM, and FIM are comprised of 4-items each and are estimated to take 5 min to complete each measure (Weiner et al. 2017). Responses range from “completely disagree” to “completely agree” and are scored from 1 to 5. Higher scores indicate greater acceptability, appropriateness or feasibility and cut-off scores are not yet available (Weiner et al. 2017).

## Statistical analysis

Using the SPPS version, 29 measures of central tendency (mean, median) and variability for continuous variables and frequency and percent for categorical variables were calculated. Variability estimates of change from baseline to post intervention for outcome measures without *p*-values were also assessed.

## Qualitative analysis

Post intervention interviews were transcribed verbatim and reviewed for accuracy by the primary investigator. Participant suggestions were categorized into 1 of 3 major categories (content, organization, terminology), which were developed during the initial intervention adaptation (Layne et al. 2024). Twenty percent of interview recordings and transcripts were validated for accuracy by a co-investigator. Guided by prior work during the initial intervention adaptation, qualitative data were categorized into positive or constructive feedback. The same themes identified in prior work were applied to these results. One additional category was identified to capture suggestions related to intervention delivery. The principal investigator assigned preliminary categories and prioritization levels to all constructive feedback. Four possible prioritization levels were utilized, including 1) high importance, adds clarity, 2) high importance, adds confusion, 3) low importance, adds clarity and 4) low importance and adds confusion. Two co-investigators reviewed the assigned preliminary categories and prioritization, and consensus was reached by the research team on final assigned categories and prioritization.

## Results

**Figure 1.**
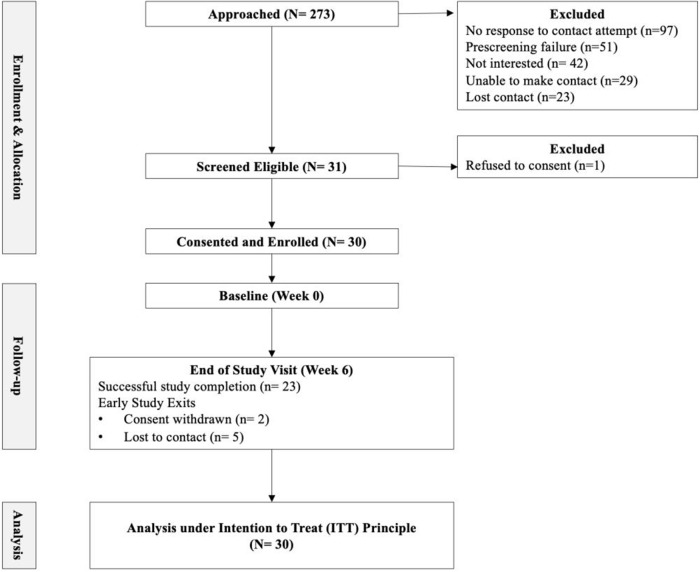
CONSORT Diagram.

A total of 273 individuals were approached regarding study participation of which 31 dyads were screened eligible. Twenty-eight dyads and two individual CG were successfully consented and enrolled into the study. Participant characteristics are provided in Table 1. Many of the care recipients were male (70%) with a mean age of 75.1 (SD = 8.3) years, compared to most CG being female (96%), spouses (85%) with a mean age of 69.5 (SD = 10.3) years. This sample was educated with majority of CG (81%) and care recipients (93%) attending at least some college. Greater than half of the caregiver participants reported having prior experience as CG. Study completion rate was 76% (21 dyads and 2 CG) successfully completed study activities (see Figure 1 for CONSORT diagram). Consent was withdrawn by 2 dyads due to time constraints and the research team lost contact with 5 dyads. No adverse events were reported.

## Feasibility outcomes

Mean post intervention scores on the Acceptability of Intervention (AIM), Intervention Appropriateness Measure (IAM) and Feasibility of Intervention (FIM) measure scores from CG (*n* = 23) were 4.36 (SD 0.5), 4.33 (SD 0.58), and 4.24 (SD 0.70) on a scale of 1–5, respectively. Post intervention mean scores for care recipients (*n* = 11) for acceptability (*n*-11), appropriateness (*n* = 13), and feasibility (*n* = 11) were 3.51 (SD = 1.18), 4.05 (SD = 0.71), and 4.05 (SD = 0.75), respectively. These scores indicate care recipients and CG found the intervention acceptable, appropriate, and feasible. On average, across the 6 week intervention period, majority of caregiver participants (58%) reported spending 30 min or less reviewing the intervention booklet. Similarly, most care recipients (55%) reported on average spending 30 min or less reviewing the intervention materials.

## Preliminary impact on knowledge and health outcomes

Minimal change from preintervention (baseline) assessments to post intervention (Week 6) was noted across health outcomes. Disease related knowledge remained relatively stable from preintervention to post intervention for CG (means preintervention: 25.18, post intervention 25.61, mean difference 0.26); however, a slight increase was noted for PLWD (means preintervention: 20.36, post intervention 22.15, mean difference 1.15) on the Alzheimer’s Disease Knowledge scale. Perceived stress and quality of life remained relatively unchanged preintervention and post intervention for both CGs and PLWD. Table 3 includes descriptive statistics for outcome variables by participant type including mean, standard deviation, change from baseline and 95% confidence intervals. Slight improvements were noted in caregiver self-efficacy (means preintervention: 6.67 and post-intervention 7.43, mean difference 0.54), unmet needs (means pre intervention; 7.93 and post intervention: 4.17, mean difference −2.39). These findings suggest that caregiver self-efficacy increased slightly while caregiver unmet needs decreased slightly as well.

**Table 3.** Mean (± standard deviation [SD]) baseline, week 6 scores, and change from baseline (calculated as baseline value minus week 6 value with 95% confidence interval [95% CI]) for participants by role

	Baseline		Week 6		Difference		
Instrument/Measure							
Caregivers	NN	Mean ± SD	N	Mean ± SD	NN	Mean ± SD	95% CI for difference
Alzheimer’s Disease Knowledge Scale	28	25.18 ± 2.45	23	25.61 ± 2.13	23	0.26 ± 1.74	−0.49 1.01
Perceived Stress Scale	28	20.50 ± 4.51	23	20.17 ± 3.35	23	−0.09 ± 3.18	−1.46, 1.29
Quality of Life in Alzheimer’s Disease (proxy)	28	34.61 ± 7.26	23	34.70 ± 6.90	23	−0.74 ± 3.73	−2.35, 0.87
Caregiver self-efficacy	28	6.67 ± 1.70	23	7.43 ± 1.54	23	0.54 ± 1.02	0.10, 0.98
Safety assessment scale	28	30.32 ± 5.98	23	30.43 ± 5.12	23	0.09 ± 4.17	−1.71, 1.89
Zarit burden interview	28	18.21 ± 9.19	23	16.00 ± 8.39	23	−1.09 ± 5.02	−3.26, 1.08
Physical Function	13	46.43 ± 11.56	15	48.28 ± 8.36	10	−1.42 ± 8.65	−7.61, 4.77
Anxiety	17	53.02 ± 9.51	18	51.84 ± 7.00	14	1.18 ± 6.87	−2.79, 5.15
Depression	13	50.90 ± 9.05	16	49.04 ± 9.44	10	−0.25 ± 6.73	−5.07, 4.56
Fatigue	19	52.04 ± 10.11	18	50.97 ± 9.40	15	−0.22 ± 10.71	−6.15, 5.71
Sleep	27	53.81 ± 4.03	23	52.26 ± 2.61	20	−2.01 ± 4.10	−3.92, −0.09
Social participation	23	38.61 ± 8.99	20	43.13 ± 12.51	19	2.20 ± 10.36	−2.79, 7.20
Pain Interference	15	51.02 ± 9.59	15	51.99 ± 7.36	13	1.74 ± 8.50	−3.40, 6.87
Person with Alzheimer’s disease							
Alzheimer’s Disease Knowledge Scale	22	20.36 ± 5.38	13	22.15 ± 5.00	13	1.15 ± 3.80	−1.57, 3.88
Perceived Stress Scale	22	16.18 ± 5.91	13	16.85 ± 4.39	13	1.38 ± 6.93	−2.81, 5.58
Quality of Life in Alzheimer’s Disease (self)	22	39.23 ± 7.35	13	41.54 ± 5.14	13	−0.08 ± 6.40	−3.94, 3.79
Digital health literacy	21	49.14 ± 17.80	13	53.92 ± 16.35	13	2.54 ± 9.81	−3.39, 8.47
Physical Function	12	40.47 ± 13.55	8	47.85 ± 8.52	5	−3.56 ± 11.69	−18.07, 10.95
Anxiety	9	53.18 ± 8.40	10	46.46 ± 16.76	5	−10.60 ± 26.61	−43.64, 22.44
Depression	14	50.69 ± 9.61	11	47.23 ± 9.45	8	−2.25 ± 7.64	−8.64, 4.14
Fatigue	15	47.49 ± 9.05	10	49.83 ± 10.08	7	4.13 ± 12.84	−7.75, 16.01
Sleep	10	46.24 ± 10.26	9	49.13 ± 5.36	7	2.84 ± 6.01	−2.71, 8.40
Social participation	3	33.66 ± 13.49	10	27.13 ± 13.79	7	44.87 ± 20.42	−63.43, 66.29
Pain Interference	10	51.02 ± 9.59	7	44.87 ± 20.42	5	0.74 ± 11.01	25.98, 63.76

## Identified challenges during intervention delivery and data collection

Two primary challenges encountered during completion of this study included scheduling conflicts for the nurse interventionist meetings and challenges completing some of the requested questionnaires. Average time between nurse interventionist meetings was 31 days (SD = 16 days) with a range between 21 days and 80 days. Meeting length for the first nurse interventionist meeting was on average 53 min (SD = 10 min) with a range of 30–65 min. Meeting length for the second nurse interventionist meeting was 39 min (SD = 15 min) with a range between 5 and 63 min. Three outliers were identified for days between meetings, all of which required multiple rescheduling attempts due to conflicts with originally selected meeting dates.

Both caregiver and care recipient participants did not consistently complete all PROMIS measures. Specifically, pain interference and physical function were completed by less than half of the participants in each group. Additionally, CG did not consistently complete the anxiety measure while care recipients did not consistently many of the PROMIS measures. Upon further discussion during the post intervention interviews, it was noted that both groups did not believe these items were applicable to their current situations.

## Post intervention interview analysis

Across 10 interviews (mean: 39 min; range 26–56 min), CG and care recipients shared 70 suggestions for revisions and 36 positive comments regarding the intervention and delivery process. Majority of suggestions received were related to content (56%), while the remaining suggestions were related to terminology (23%), organization (16%), and delivery (6%). Most suggestions were only mentioned a single time; however, there were 2 suggestions mentioned by multiple participants including creation of an index and table of contents. Most suggestions related to content were related to enhancements to clarify content related to understanding the disease (32%), self-management strategies for the PLWD (21%), or strategies for CG (15%). The remaining suggestions for content revisions were related to resources (12%), planning ahead (12%), and safety (9%). Table 4 provides initial coding categories and sample participant suggestions.

**Table 4.** Initial coding categories and sample participant suggestion

Category	Participant suggestions
**Content**	
Understanding the disease	Include more information on what to expect as the disease progresses
Self-management	Include possible side effects for medications used to manage symptoms
Strategies for caregiving	Consider adding a checklist for caregivers to know when they might need help
Resources	Would have liked more information on support groups specifically for the care recipient
Planning ahead	Consider adding information on brain banks
Safety	Add information about medication reconciliation
**Organization**	Have a section for all the resources to be centrally located
	Create a subject index with all applicable links in 1 location
**Terminology**	Remove the term “gets worse” as it has a negative connotation
	Clarify what “reducing stimulation” means
**Delivery**	Consider having separate books for the caregiver and care person with dementia, so that content not relevant to the individual is removed
	Consider having an electronic version with clickable links as a complement to the paper booklet

Participants agreed that having periodic visits to reinforce content from intervention materials was helpful. When asked about the possibility of adding a social support component to the intervention, most participants agreed this would be a helpful addition. Finally, we also asked participants about whether including content on physical activity, healthy aging, and stress management would be beneficial, and all participants endorsed adding this type of content would add value to the intervention.

## Discussion

The primary aim of this study was to examine feasibility and acceptability of SUPPORT-D^TM^ for use with persons living with mild cognitive impairment or Alzheimer’s disease and their CG. Results indicate that both CG and care recipients found the intervention to be feasible and acceptable. Identified challenges during study implementation included scheduling conflicts for attendance at nurse interventionist meetings and incomplete data collection, specifically with PROMIS measures. Care coordination has been established as a challenging aspect of caregiving specifically for those living with dementia. This is a likely explanation for difficulty scheduling and keeping established nurse interventionist appointments.

Miranda and colleagues (Miranda et al. 2019) identified that limited high quality palliative care interventions exist for community dwelling persons with dementia. Similarly, Walsh and colleagues examined palliative care interventions for those living with advanced dementia and results were inconclusive regarding how palliative care can best be used (Walsh et al. 2021). Evidence suggests organizational changes in care delivery may improve comfort in dying and ACP interventions may improve documentation of advanced directives and discussions of goals of care potentially increasing goal concordant care (Walsh et al. 2021). Early planning including palliative care has the potential to mitigate future stress, improve quality of life, and ensure person and family centered care for those living with dementia and their CG (Whitlatch and Orsulic-Jeras 2018).

Anticipatory guidance has been identified as an important component coupled with care navigation skills for PLWD and CG (Shafir et al. 2022; Cole et al. 2023). However, limited interventions which are focused on or include anticipatory guidance exist. Shafir and colleagues (Shafir et al. 2022) proposed 6 topics to include as part of anticipatory guidance for those living with dementia and CG including: 1) asking for permission and addressing preferences, sharing expected disease course and prognosis, normalizing experiences, shifting focus of management as disease progresses, providing opportunities for CG to speak with clinicians separately from care recipients and assisting with ACP including document completion. A Program of SUPPORT-D^TM^ aligns well with this suggested framework and has potential to aid clinicians in providing anticipatory guidance to those living with cognitive impairment.

## Strengths and limitations

Strengths of this study included integration of quantitative and qualitative measures of feasibility and integration of both caregiver and care recipient perspectives. While this study was not powered to detect improvements in outcomes, post intervention improvements were noted for caregiver self-efficacy and unmet needs. It is important to note the somewhat homogenous small sample not uncommon in pilot and feasibility studies. Nonetheless, these results provide important context for designing a subsequent larger randomized trial to examine the effectiveness of SUPPORT-D^TM^ in improving caregiver self-efficacy and reducing unmet needs.

## Conclusion

Caregiver and care recipients reported a Program of SUPPORT-D^TM^ was acceptable and feasible. Future revisions to the intervention are planned based on the recommendations provided during the post-intervention interviews. Based on the findings from this study, we are planning to further optimize the intervention by incorporating and testing additional intervention components regarding physical activity/stress management and social support using a factorial design. Additionally, future work examining the feasibility of integrating a Program of SUPPORT-D^TM^ into primary care delivery for those suspected of or diagnosed with cognitive impairment is also planned.

## References

[ref1] ADKS | Clinical Geropsychology Laboratory | Washington University in St. Louis (n.d.). https://sites.wustl.edu/geropsychology/adks/ (accessed 7 September 2023).

[ref2] Alzheimer’s Association (2024) Alzheimer’s Association 2024 Alzheimer’s Disease Facts and Figures. 10.1002/alz.13809 PMC11095490 38689398

[ref3] Arvanitakis Z, Shah RC and Bennett DA (2019) Diagnosis and management of dementia: a review. *JAMA* 322(16), 1589–1599. 10.1001/jama.2019.4782 31638686 PMC7462122

[ref4] Bédard M, Molloy DW, Squire L, et al. (2001) The Zarit Burden interview: a new short version and screening version. *The Gerontologist* 41(5), 652–657. 10.1093/geront/41.5.652 11574710

[ref5] Black BS, Johnston D, Rabins PV, et al. (2013) Unmet needs of community-residing persons with dementia and their informal caregivers: findings from the maximizing independence at home study. *Journal of the American Geriatrics Society* 61(12), 2087–2095. 10.1111/jgs.12549 24479141 PMC4001885

[ref6] Butler M, Gaugler JE, Talley KMC, et al. (2020) Care Interventions for People Living With Dementia and Their Caregivers. Agency for Healthcare Research and Quality (AHRQ). https://effectivehealthcare.ahrq.gov/products/care-interventions-pwd/report (accessed 5 September 2023).

[ref7] Carpenter BD, Balsis S, Otilingam PG, et al. (2009) The Alzheimer’s disease knowledge scale: development and psychometric properties. *The Gerontologist* 49(2), 236–247. 10.1093/geront/gnp023 19363018 PMC2667675

[ref8] Center to Advance Palliative Care (2024) Palliative Care Definition | What is Palliative Care. https://www.capc.org/about/palliative-care/ (accessed 5 September 2023).

[ref9] Clague F, Mercer SW, McLean G, et al. (2017) Comorbidity and polypharmacy in people with dementia: insights from a large, population-based cross-sectional analysis of primary care data. *Age and Ageing* 46(1), 33–39. 10.1093/ageing/afw176 28181629

[ref10] Cohen S, Kamarck T and Mermelstein R (1983) A global measure of perceived stress. *Journal of Health and Social Behavior* 24(4), 385. doi: 10.2307/2136404 6668417

[ref11] Cole CS, Dafoe A, Tietbohl CK, et al. (2023) Care challenges of home health patients living with dementia: a pathway forward with palliative care. *BMC Palliative Care* 22(1), 122. doi: 10.1186/s12904-023-01247-9 37641096 PMC10464392

[ref12] de Courval LP, Gélinas I, Gauthier S, et al. (2006) Reliability and validity of the safety assessment scale for people with dementia living at home. *Canadian Journal of Occupational Therapy* 73(2), 67–75. doi: 10.1177/000841740607300201 16680910

[ref13] Eskildsen A, Dalgaard VL, Nielsen KJ, et al. (2015) Cross-cultural adaptation and validation of the Danish consensus version of the 10-item perceived stress scale. *Scandinavian Journal of Work, Environment & Health* 41(5), 486–490. doi: 10.5271/sjweh.3510 26111225

[ref14] Fox S, FitzGerald C, Harrison Dening K, et al. (2017) Better palliative care for people with a dementia: summary of interdisciplinary workshop highlighting current gaps and recommendations for future research. *BMC Palliative Care* 17(1), 9. doi: 10.1186/s12904-017-0221-0 28705196 PMC5512895

[ref15] Harris PA, Taylor R, Thielke R, et al. (2009) Research electronic data capture (REDCap)–a metadata-driven methodology and workflow process for providing translational research informatics support. *Journal of Biomedical Informatics* 42(2), 377–381. doi: 10.1016/j.jbi.2008.08.010 18929686 PMC2700030

[ref16] Hashimie J, Schultz SK and Stewart JT (2020) Palliative care for dementia. *Clinics in Geriatric Medicine* 36(2), 329–339. doi: 10.1016/j.cger.2019.11.011 32222305

[ref17] Hays RD, Spritzer KL, Schalet BD, et al. (2018) PROMIS®-29 v2.0 profile physical and mental health summary scores. *Quality of Life Research : An International Journal of Quality of Life Aspects of Treatment, Care and Rehabilitation* 27(7), 1885–1891. doi: 10.1007/s11136-018-1842-3 29569016 PMC5999556

[ref18] Jennings LA, Palimaru A, Corona MG, et al. (2017) Patient and caregiver goals for dementia care. *Quality of Life Research : An International Journal of Quality of Life Aspects of Treatment, Care and Rehabilitation* 26(3), 685–693. doi: 10.1007/s11136-016-1471-7 28000094 PMC5382930

[ref19] Kahle-Wrobleski K, Ye W, Henley D, et al. (2016) Assessing quality of life in Alzheimer’s disease: implications for clinical trials. *Alzheimer’s & Dementia : Diagnosis, Assessment & Disease Monitoring* 6, 82–90. doi: 10.1016/j.dadm.2016.11.004 PMC5312555 28229126

[ref20] Khutok K, Janwantanakul P, Jensen MP, et al. (2021) Responsiveness of the PROMIS-29 scales in individuals with chronic low Back pain. *Spine* 46(2), 107. doi: 10.1097/BRS.0000000000003724 33347091

[ref21] Kim H, Cho J, Park WS, et al. (2021) Characteristics of advance care planning interventions across dementia stages: a systematic review. *Journal of Nursing Scholarship* 53(2), 180–188. doi: 10.1111/jnu.12624 33476479

[ref22] Layne D, Milano N, Kelechi T, et al. (2024) Preliminary findings of an adapted nurse-led palliative care intervention. *Journal of Palliative Medicine* 27(1), 56–62. doi: 10.1089/jpm.2023.0324 37819751 PMC10790543

[ref23] Lindell KO, Klein SJ, Veatch MS, et al. (2021) Nurse-led palliative care clinical trial improves knowledge and preparedness in caregivers of patients with idiopathic pulmonary fibrosis. *Annals of the American Thoracic Society* 18(11), 1811–1821. doi: 10.1513/AnnalsATS.202012-1494OC 34003726 PMC8641836

[ref24] Miranda R, Bunn F, Lynch J, et al. (2019) Palliative care for people with dementia living at home: a systematic review of interventions. *Palliative Medicine* 33(7), 726–742. doi: 10.1177/0269216319847092 31057088 PMC6620864

[ref25] Murphy E, Froggatt K, Connolly S, et al. (2016) Palliative care interventions in advanced dementia. *The Cochrane Database of Systematic Reviews* 2016(12), 1–48. doi: 10.1002/14651858.CD011513.pub2 PMC6463843 27911489

[ref26] Ritter PL, Sheth K, Stewart AL, et al. (2022) Development and evaluation of the eight-item caregiver self-efficacy scale (CSES-8). *The Gerontologist* 62(3), e140–e149. doi: 10.1093/geront/gnaa174 33146727 PMC8963154

[ref27] Sclan SG and Reisberg B (1992) Functional assessment staging (FAST) in Alzheimer’s disease: reliability, validity, and ordinality. *International Psychogeriatrics* 4(3), 55–69. doi: 10.1017/S1041610292001157 1504288

[ref28] Shafir A, Ritchie CS, Garrett SB, et al. (2022) “Captive by the uncertainty”—experiences with anticipatory guidance for people living with dementia and their caregivers at a specialty dementia clinic. *Journal of Alzheimer’s Disease* 86(2), 787–800. doi: 10.3233/JAD-215203 PMC9717709 35124641

[ref29] Stewart JT and Schultz SK (2018) Palliative care for dementia. *Psychiatric Clinics of North America* 41(1), 141–151. doi: 10.1016/j.psc.2017.10.011 29412842

[ref30] Tilburgs B, Vernooij-Dassen M, Koopmans R, et al. (2018) Barriers and facilitators for GPs in dementia advance care planning: a systematic integrative review. *PLoS ONE* 13(6), e0198535. doi: 10.1371/journal.pone.0198535 29924837 PMC6010277

[ref31] Torisson G, Stavenow L, Minthon L, et al. (2016) Reliability, validity and clinical correlates of the quality of life in Alzheimer’s disease (QoL-AD) scale in medical inpatients. *Health and Quality of Life Outcomes* 14, 90. doi: 10.1186/s12955-016-0493-8 27301257 PMC4908755

[ref32] van der Vaart R and Drossaert C (2017) Development of the digital health literacy instrument: measuring a broad spectrum of health 1.0 and health 2.0 skills. *Journal of Medical Internet Research* 19(1), e27. doi: 10.2196/jmir.6709 28119275 PMC5358017

[ref33] Van Ooteghem K, Musselman KE, Mansfield A, et al. (2019) Key factors for the assessment of mobility in advanced dementia: a consensus approach. *Alzheimer’s & Dementia: Translational Research & Clinical Interventions* 5, 409–419. doi: 10.1016/j.trci.2019.07.002 31508479 PMC6726753

[ref34] Walsh SC, Murphy E, Devane D, et al. (2021) Palliative care interventions in advanced dementia. *The Cochrane Database of Systematic Reviews* 2021(9), CD011513. doi: 10.1002/14651858.CD011513.pub3 PMC8478014 34582034

[ref35] Weiner BJ, Lewis CC, Stanick C, et al. (2017) Psychometric assessment of three newly developed implementation outcome measures. *Implementation Science : IS* 12, 108. doi: 10.1186/s13012-017-0635-3 28851459 PMC5576104

[ref36] Whitlatch CJ and Orsulic-Jeras S (2018) Meeting the informational, educational, and psychosocial support needs of persons living with dementia and their family caregivers. *The Gerontologist* 58(suppl_1), S58–S73. doi: 10.1093/geront/gnx162 29361068

